# Engaging Remote Aboriginal Communities in COVID-19 Public Health Messaging *via* Crowdsourcing

**DOI:** 10.3389/fpubh.2022.866134

**Published:** 2022-05-13

**Authors:** Miriam Glennie, Michelle Dowden, Mark Grose, Meg Scolyer, Alessandra Superina, Karen Gardner

**Affiliations:** ^1^Public Sector Research Group, University of New South Wales, Canberra, ACT, Australia; ^2^One Disease, Darwin, NT, Australia; ^3^Skinnyfish, Darwin, NT, Australia

**Keywords:** crowdsourcing, remote Aboriginal communities, health communication, COVID-19, social media, Indigenous language

## Abstract

Health comunication is a critical component of pandemic mitigation, but mainstream prevention messaging often lacks social, cultural and linguistic relevance to vulnerable populations. This community case study presents a novel, highly participatory pandemic prevention communication campaign that engaged individuals in remote Aboriginal communities of the Northern Territory of Australia directly in prevention messaging via crowdsourcing, and distributed videos to remote area post-codes via targeted Facebook advertising. Facebook metrics, administrative campaign data and national statistics are used to assess campaign reach and engagement. The case study discusses lessons learned from the campaign, including how seeking unscripted COVID-19 prevention video messaging can support community ownership of pandemic messaging, rapid content generation, and a high level of Facebook user engagement. It also discusses the effectiveness of targeting remote area post-codes *via* Facebook advertising both to reach the target audience, and to support quality improvement assessments to inform health communication decision-making in a low resource setting.

## Introduction

Aboriginal and Torres Strait Islander Australians are at higher risk of morbidity and mortality associated with pandemics than other Australians due to a range of factors associated with limited access to health care and high rates of non-communicable disease. During the 2009 H1N1 pandemic, indigenous communities in Australia, New Zealand and Canada were over-represented in the number of severe cases requiring hospitalization and among fatal cases (1). In the Northern Territory (NT) of Australia, Aboriginal and Torres Strait Islander Australians were hospitalized at a much higher rate than in other parts of Australia (2). In remote Aboriginal communities, factors associated with limited access to health care and basic essentials together with substandard housing, overcrowding, and racism compound this risk (3).

Prevention information is a critical component of pandemic mitigation, especially for groups who are at high risk such as Aborignal and Torres Strait Islander populations. Prevention messaging should be rapid and explicit with clear information that aligns with local culture and values, and that reflects day to day community life, and presents health information in local Aboriginal languages (4–6).

Driven by an aim to facilitate community ownership of pandemic messaging, rapidly generate localized content, and faced with remote Aboriginal community closures and travel restrictions, *One Disease*-an NT based, health-related non-profit that works with remote Aboriginal communities-undertook a novel pandemic prevention messaging campaign. The organization partnered with a local music production company, *Skinnyfish*, to conduct a crowdsourcing campaign that sought COVID-19 prevention videos directly from individuals in remote communities. Accepted videos were then distributed to remote area post-codes via targeted Facebook advertising.

This community case study draws on Facebook metrics, administrative data from the campaign, copies of the videos posted on the *One Disease* and *Skinnyfish* public Facebook pages, and national population statistics to assess campaign reach and user engagement at both campaign and individual ad levels. It discusses the lessons learned from this analysis, which was designed to inform continuous improvement processes at *One Disease*, and may support health communication planning in comparable organizations and/or contexts.

This paper presents the context of the campaign before outlining the rationale for the campaign in light of contextual factors. It then describes the design and implementation of the campaign before analyzing its results in terms of reach and engagement. Lessons learned are then discussed before concluding with study limitations.

## Context

The Northern Territory of Australia covers >1.3 million square kilometers of central northern Australia. It has a population of 247,000 of which ~30% are Aboriginal and Torres Straight Islanders, compared with ~3% of the total Australian population (ABS unpublished census statistics). There are hundreds of small Aboriginal communities in remote regions of the NT, ranging in population size from a few thousand to less than one hundred, and over 100 Aboriginal languages are spoken (7).

A history of colonization and discrimination against Aboriginal and Torres Strait Islander Australians has impacted the social determinants of health and contributed to higher rates of communicable and non-communicable diseases, and reduced life expectancy, which is more than 5 years lower than in non-Indigenous populations (8). Fifty percent of Aboriginal and Torres Strait Islander adults have a chronic disease and a quarter have co-morbidities, particularly among those aged over fifty. In remote communities, factors associated with limited access to health care and basic essentials together with substandard housing and overcrowding underpin elevated health risk (9). Many remote communities are hundreds of kilometers from healthcare facilities.

A network of Aboriginal Community Controlled Health Organisations (ACCHOs) provide primary healthcare to Aboriginal and Torres Strait Islander Australians throughout the NT, including in remote Aboriginal communities. Early in the COVID-19 pandemic, Indigenous leaders and ACCHOs took swift action to call for remote community closures to limit the flow of people in and out, and to work with government to establish a strategic response and workforce preparedness. Throughout the pandemic, Australia's international borders have remained largely closed, as have many state and territory borders to internal movement. The NT has had some of the strictest border control policies in the country.

The pandemic prevention messaging communicated by the Australian Federal government in early stages of the pandemic was English language only, and lacked cultural relevance and practical applicability in Aboriginal and Torres Strait Islander communities. It was this public health communication gap that *One Disease* sought to address with its crowdsourced social media campaign.

## Rationale for the Campaign

Public health communication campaigns have often drawn on relatively superficial community engagement through focus groups or interviews, and typically require long consultation periods (10, 11). The COVID-19 pandemic spurred innovation in methods of public health communication, as neither in-person community engagement nor extended co-design or consultation processes have been feasible given the urgency of the information need, limitations on travel and social distancing requirements. In the NT, many community based ACCHOs have been active in rapidly developing Indigenous language COVID-19 prevention communications in partnership with local communities (6, 12).

*One Disease* sought to trial another participatory approach to Indigenous language prevention messaging, that is suitable for rapid content generation, by seeking unscripted videos directly from individuals in remote communities via a crowdsourcing campaign. *One Disease* is a not-for-profit health organization that aims to eliminate crusted scabies, a communicable skin disease prevalent in remote Aboriginal communities, as a public health concern. Following the outbreak of COVID-19 in Australia, *One Disease* was unable to continue its physical outreach work in remote communities due to travel restrictions and community lock-downs. As a result, the organization decided to direct some funds toward a crowdsourcing campaign for COVID-19 pandemic prevention. The organization wanted to continue to contribute to the health of the communities with which it works and to maintain its relationship with remote communities when unable to travel. *One Disease* also saw an opportunity to trial a new approach to participatory health promotion that, if effective, could be used in future work on communicable skin disease.

Crowdsourcing, which involves the outsourcing of an organizational function or task to a crowd, is increasingly popular in health service delivery, as involving individuals from the intended audience of a health initiative in problem solving and solution ideation can generate more relevant and acceptable content (12–14). In health, the most common uses of crowdsourcing are for creating collaborative communities and accessing a dispersed labor force (15). Healthcare organizations may turn to crowds when seeking input from a group of individuals defined by specific health or demographic characteristics, or by specific skills (e.g., technical, information processing). The effectiveness of crowdsourcing has been evidenced in many areas of health research and practice, including diagnosis, surveillance, and study recruitment (14–17), and crowdsourced health promotion videos have been found equally as effective as healthcare provider generated videos (18).

Crowdsourcing has been used widely during the COVID-19 pandemic, in particular for engaging the public in large scale surveillance, and for engaging dispersed health experts in collective problem solving (19, 20). There is no known research on crowdsourcing in Aboriginal and Torres Strait Islander communities for pandemic mitigation specifically, or health promotion more generally. Crowdsourcing has, however, been used in other settings to engage indigenous peoples in collaborative communities–for example the collation of threatened language databases, or cultural heritage collections (21–23), as well as in more transactional, labor market crowdsourcing such as ecotourism mapping (24).

Beyond establishing a method for supporting locally generated, language content, *One Disease* needed a medium to distribute video content to the target audience. Facebook is used widely by Aboriginal and Torres Strait Islander Australians, and many health services, including ACCHOs and other non-profit organizations use Facebook to disseminate health promotion material to Aboriginal and Torres Strait Islander communities (25, 26). However, existing studies of health promotion campaigns distributed *via* Facebook have reported on content being posted on a health service organisation's profile page, not distributed via advertising.

One limitation of relying on profile page posts to disseminate health promotion information is that it relies on the online social network of the healthcare organization, which can limit reach. Facebook advertising can reach a much larger population, is relatively low cost and can support the targeting of specific populations using user data. *One Disease* also saw value in Facebook advertising from a quality improvement perspective, as it allows for easier and more granular evaluation than other forms of media advertising such as TV; the latter generates only aggregate, estimated reach data, while Facebook generates granular data on reach to unique users, total number of views, as well as length of view for video content.

One means through which populations can be targeted in Facebook advertising is via the post-code in which a user is located. In areas in which ethnic groups are concentrated geographically, post-code can be a proxy for ethnic background. In the US, post-code (zip-code) targeting has been used to target African Americans and Latinos with tailored health promotion content (27). There is no known research on post-code targeted Facebook advertising in remote Aboriginal communities. The targeting approach was selected for this campaign on the basis of its potential to reach users in remote communities.

## Campaign Implementation

The crowdsourcing campaign was funded by *One Disease* and facilitated on a pro bono basis by *Skinnyfish*–a local music production company that works with Aboriginal and Torres Strait Islander artists. Both organizations have extensive social networks and working relationships in remote Aboriginal communities in the NT. Two crowdsourcing rounds were conducted, each with 2 week crowdsourcing periods and 3 week advertising periods during the peak of the first wave of COVID-19 in Australia.

Distribution of an invitation to script, design and produce COVID-19 prevention videos in local Aboriginal languages was facilitated via phone contact by *Skinnyfish* on behalf of *One Disease*. The director of *Skinnyfish* initially invited groups or individuals living in remote communities who had experience generating community messaging or experience in the entertainment industry. A strategy for contacting others in the community was developed in accordance with cultural ways, including communicating with and extending the invitation to females.

The brief given to potential contributors was to generate a video presenting a clear and simple message in a local Aboriginal language to regularly wash hands for at least 20 s (first campaign), or to maintain 1.5 m physical distancing (second campaign). No scripting was provided. To maintain authenticity and respect for the contributors, no post-production editing was conducted, so contributors knew that their work would not be modified. Videos were required to be at least 30 s long, and could be submitted for consideration via WhatsApp message to the director of *Skinnyfish*.

The acceptability criteria for videos were a clear message about one or both of the two COVID-19 hygiene topics, and an acceptable video length (i.e., minimum 30 s). Imperfect videography was expected and accepted. All videos were filmed on mobile phones. Videos were assessed by a panel of *One Disease* staff, all public health nurses, to ensure that demonstrations and/or examples presented were accurate from a public health perspective. If deemed inaccurate, contributors were provided feedback and given the opportunity to submit a revised video. Upon confirmation of acceptance, contributors were paid the equivalent of one full day's work.

A total of 19 videos were received, of which 18 were accepted. One video was rejected on the basis that it did not present a clear COVID-19 prevention message. Of the 18 accepted videos, there were nine videos about handwashing, six about physical distancing and three about both. Six Aboriginal languages were spoken across the video collection, with many videos repeating the key message in English. All accepted videos were produced by local musicians, entertainers or known community figures.

The accepted videos involved either a demonstration or description of COVID-19 prevention practices applied to the local community setting. Handwashing demonstrations were presented in local community settings such as a home, recreation center or outdoor communal space. Physical distancing demonstrations were either in commercial settings (e.g., bank, taxi), or social community settings (e.g., an informal contact free drop-off grocery shopping, and not sharing drinks or handshakes).

On completion of the crowdsourcing campaign, *One Disease* contracted a local advertising firm to distribute the videos via Facebook paid advertising. Australian Bureau of Statistics [ABS] data was used to identify postcodes relevant to the languages spoken in the videos; the advertising campaign was then set up to target these postcodes. The campaign was set up to prioritize ThruPlays–the term used by Facebook to describe video views of 15 s or more; ThruPlays were prioritized to maximize the likelihood of the videos being viewed in full, and subsequently maximizing exposure to the videos' key message.

The remote postcodes targeted in the distribution strategy, along with the relevant languages from the campaign, the population size and the percentage of the population from Aboriginal or Torres Straight Island (ATSI) backgrounds [compared with ~3% in total Australian population (ABS unpublished census data)] are presented in Table 1.

**Table 1:** Postcodes and populations.

**Postcode**	**Region**	**Languages**	**Population**	**% ATSI**
0822	NT top end central	Djambarrpuyngu, Kunwinjku, Ndjébbana	25,558	70%
0880	NT top end west	Djambarrpuyngu	5,212	43%
0872	NT south and nearby WA	Western Arrente	15,468	80%
0852	NT central	Kriol, Anindilyakwa	8,300	72%

For the first week of the handwashing campaign, and for two of the commercial setting physical distancing clips, videos were also distributed in Darwin (the capital city). At the end of the second campaign, one edited music video clip about handwashing was made in English by one of the first campaign contributors and *Skinnyfish*, and distributed across the NT and area of Western Australia with shared postcode.

## Results

### Reach

Campaign level Facebook metrics are presented in Table 2. Campaign reach was assessed using Facebook's ‘Reach' measure, which captures the number of unique users exposed to the ad. The campaign reached 91,295 Facebook users in a population (aged 15+) of 167,277 in the NT plus part of WA. On the assumption that each Facebook user is an individual, this represents 55% of the population.

**Table 2:** Facebook metrics.

**Metric**	**Result**
Reach	91,295
Impressions	638,294
ThruPlays	75,591
Post engagement	233,535
Cost per Thruplay	$0.03
Cost per post engagement	$0.01

Impressions is a Facebook measure of the number of times a video has been displayed. Over the 2 x 3 week distribution periods, a campaign video was displayed to a user 638,294 times. Although it is not possible to disaggregate to individual users, as an indication of frequency of exposure, with a reach of 91,295 individual users, this represents 6.99 impressions per user. ThruPlays of 75,591 represent 0.83 full video views per user. In reality, Impression and ThruPlay numbers may be much higher for some users than others, but these aggregate numbers provide an overall indication of exposure.

In comparison to a study assessing the reach of health promotion posts on the Facebook profile page of ACCHOs in comparable communities, the reach achieved via Facebook advertising appears much higher. Hefler et al. (26) reported an average reach of 248 users per post in their study, while the average reach for each video in the targeted Facebook advertising campaign reported in this study was 5,370. That this high level of reach can be achieved at relatively a low cost of $0.01 per engagement (view or reaction) suggests that post-code targeted Facebook advertising may be a useful distribution strategy for stand alone campaigns in low resource settings.

### Engagement

To compliment campaign level analysis, Reach and ThruPlay metrics were analyzed for individual ads to identify which video features may support user engagement and support quality improvement in *One Disease* public health communication. Engagement with individual ads was assessed by the number of ThruPlays as a percentage of the number of users reached; this figure was used to rank the engagement levels for individual videos. Content analysis was conducted to identify features on individual videos; this involved coding based on visual observation of videos for presenter age category (child, youth, adult, older adult) and gender, video setting, activities, other features such as music or humor. Video language and postcode(s) distributed to were also recorded. These details are presented in Table 3 presents.

**Table 3:** Individual ad content, language, postcode, reach and ThruPlays.

**Language**	**Postcode(s)**	**Content**	**Reach**	**Thru-Plays**	**Thru-Plays (% Reach)**
**Handwashing**					
Ndjébbana	0822	Female local band members demonstrating hand washing	6,458	1374	21%
Djambarrpuyngu	0822, 0800	Male local band member talking about handwashing	654	174	27%
Djambarrpuyngu	0822, 0800	Male local band member demonstrating handwashing with kids to music	6,602	2290	35%
Djambarrpuyngu	0822, 0800	Humor and local well-known community member and also musician	724	94	13%
Djambarrpuyngu	0822, 0800	Older male clear message and direct message	1,723	269	16%
Kriol	0852	Male demonstrating and working with kids	3,277	905	28%
Western Arrente	0872	Older male well known musician clear message and direct message	1,766	304	17%
Anindilayka	0852	Local musician clear message	1,413	264	19%
Kunwinku	0822	Older local musician clear message and direct message also using local environment.	6,614	915	14%
English	Statewide	Local musician singing and kids dancing	53,758	18,192	34%
		*Skinnyfish profile page*	10,800	
**Physical distancing**					
Djambarrpuyngu	0822, 0800	Woman with kids demonstrating shopping drop-off to older man and humor	18,488	5,287	29%
Djambarrpuyngu	0822, 0800	Male talking about physical distancing no setting	3,277	911	28%
Djambarrpuyngu	0822, 0800	Older male talking about physical distancing in taxi	10,452	2,993	29%
Djambarrpuyngu	0822, 0800	Older male talking about physical distancing in home	6,168	1,669	27%
Djambarrpuyngu	0822, 0800	Older male talking about physical distancing at ATM	9,038	2,236	25%
Djambarrpuyngu	0822, 0800 +Darwin	Older male talking about physical distancing in bank	37,088	11,844	32%
Kriol	0852	Local band demonstrating with young people	2,159	905	42%
Kunwinku	0822	Older local musician clear message and direct message also using local environment.	4,436	789	18%
Western Arrente	0872	Local band singing	3,697	721	20%

Overall, content involving multiple activities or a storyline generated more engagement than spoken word alone, or straightforward demonstration (like washing hands). Videos in outdoor or commercial (shops, banks etc.) settings generated more engagement than those in homes or with no obvious setting.

Of the five most popular videos, four involved groups of children and/or young people participating in activities or demonstration in a fun or funny way. The handwashing music clip, which involved children and young people singing and dancing, had the highest number of ThruPlays of all videos in the ad campaign. It was also posted on the *Skinnyfish* public Facebook profile page; as at September 2020 it has 100,800 ThruPlays. Analysis of user comments revealed a number of individuals stating that they would share the video with children or young people in their classrooms or youth groups, which suggests the videos may be able to generate engagement beyond direct to users in Facebook.

## Discussion

These findings show that crowdsourcing can be an effective method for involving individuals in remote Aboriginal communities in public health communication. In particular, seeking unscripted COVID-19 prevention video messaging supported community ownership of pandemic messaging, and generated a high level of Facebook user engagement. The findings also show that public health messages can be distributed to individuals in remote Aboriginal communities via post-code targeted Facebook advertising at very low cost ($0.03 per ThruPlay). In campaigns seeking high levels of reach and exposure, post-code targeted advertising may represent an effective alternative to Facebook profile page posts, with the *One Disease* campaign achieving a reach many multiples higher than those reported in a comparable study (26). In seeking to understand video characteristics supportive of user engagement, Facebook advertising also facilitates more accurate and granular evaluation than traditional advertising mediums such as TV or radio. The capacity to use campaign metrics to undertake a post-campaign quality improvement study can support health communication decision-making in resource scarce settings such as non-profits.

The most viewed campaign videos displayed features known to improve engagement levels in health communication, such as a clear and simple message that holds practical value, and that is presented by an individual or group who hold social currency in the community (28). The finding that videos involving children and/or young people were amongst the most popular would require primary research to assess whether young people were the drivers of the higher view numbers, whether adult users showed videos to young people (as suggested by some teachers and youth workers in comment data), or that their involvement simply made for more engaging videos. The NT, and remote Aboriginal communities in particular, have larger youth populations than the rest of Australia (ABS unpublished statistics). Young people have been posed as conduits of information in health promotion (29) and disaster preparedness (30), and as potential change agents in households (31), but the evidence base is limited. Further research is needed to understand the role of young people as both producers and users of COVID-19 prevention messages.

The campaign also provides further evidence that community based non-profit organizations can play a valuable role in the translation of mainstream health communication through deep social networks in underserved communities (32). Although remote Aboriginal communities are serviced by a network of community controlled health organizations, this is not the case in many underserved communities internationally. This community case study shows how the strength and social currency of community based organisation's social network can support community engagement with public health campaigns, and as such, the potential benefit of involving these actors in mainstream public health communication initiatives.

## Limitations and Areas for Improvement

The high rate of acceptability in videos (18 accepted of the 19 videos submitted) and positive findings around engagement with crowdsourced videos may not be replicable with other populations. All accepted videos were generated by local musicians, entertainers or community figures who had experience with creative and/or public performance. These local figures are illustrative of the creative talent present in even small Aboriginal communities with populations in the hundreds. However, access to this creative talent was facilitated via partnership with a local music production company, which may not be present in all settings. Video quality may be lower if generated by the general population, and user engagement may be lower for videos not involving a known community figure.

The main area for improvement in campaign implementation is more balanced gender representation in videos; 17 of the 18 videos consisted solely of male presenters, or a male lead presenter. It was not appropriate for the Director of the music production company, as a male from outside the community, to make direct phone contact with women in the community; as such, invitations to participate were transmitted via male community members. Future campaigns may benefit from implementation partnerships with multiple community organizations to ensure that invitations to participate can be communicated directly to both male and female community members.

This quality improvement study of reach and engagement is limited to Facebook metrics; primary data would be needed to improve campaign evaluation and to understand engagement statistics. In the available format of Facebook metrics, it is not possible to attribute ThruPlays to specific users, meaning it is not possible to identify whether most users viewed one ad of the many they were shown, or a smaller percentage of users viewed multiple ads. This distinction would be important to accurately assess exposure and engagement. Furthermore, in examining only campaign media metrics, the study is also unable to assess whether exposure had any impact on individual or community behavior.

## Data Availability Statement

The data analyzed in this study is subject to the following licenses/restrictions: the Facebook analytics data accessed for this research are held in a commercial in confidence agreement by a digital marketing firm. Aggregate statistics were shared for the purpose of quality improvement at *One Disease*.

## Author Contributions

MGl was involved in literature review, data extraction and analysis, and led manuscript preparation. MD was involved in campaign conceptualization, design and implementation, data interpretation, and manuscript preparation. MGr was involved in campaign design and implementation and manuscript preparation. MS was involved in campaign implementation and manuscript preparation. AS was involved in campaign implementation and manuscript approval. KG was involved in literature review, data interpretation, and manuscript preparation. All authors contributed to the article and approved the submitted version.

## Funding

KG and MGl received funding for this work as part of the *One Disease* program evaluation.

## Conflict of Interest

KG and MGl receive funding from *One Disease* for the evaluation of the *One Disease* program. MD is CEO of *One Disease*. MGr was Director of Skinnyfish. The remaining authors declare that the research was conducted in the absence of any commercial or financial relationships that could be construed as a potential conflict of interest.

## Publisher's Note

All claims expressed in this article are solely those of the authors and do not necessarily represent those of their affiliated organizations, or those of the publisher, the editors and the reviewers. Any product that may be evaluated in this article, or claim that may be made by its manufacturer, is not guaranteed or endorsed by the publisher.
